# Understanding the Pathophysiology of Thrombotic APS through Animal Models

**DOI:** 10.3390/ijms22052588

**Published:** 2021-03-04

**Authors:** Alex A. Gandhi, Shanea K. Estes, Christine E. Rysenga, Jason S. Knight

**Affiliations:** Division of Rheumatology, Department of Internal Medicine, University of Michigan, Ann Arbor, MI 48109, USA; alexgand@med.umich.edu (A.A.G.); skestes@med.umich.edu (S.K.E.); crysenga@med.umich.edu (C.E.R.)

**Keywords:** antiphospholipid syndrome, antiphospholipid antibodies, thrombosis, animal models

## Abstract

Antiphospholipid syndrome (APS) is a leading acquired cause of thrombotic events, with a notable tendency to promote thrombosis in vascular beds of all sizes, including both arterial and venous circuits. While pathogenic antiphospholipid antibodies circulate at relatively stable levels in blood, thrombosis tends to manifest as discrete and acute events, suggesting the requirement for a “second hit.” While this two-hit model is generally accepted, much remains to be learned about exactly how antiphospholipid antibodies predispose to thrombosis in vivo and exactly how this predisposition interacts with the second hit. To this end, investigators have turned to animal models. Numerous approaches for modeling APS in animals have been described to date, each with potential advantages and disadvantages. This review will attempt to describe the most common APS models employed so far while discussing some pros and cons of each. Mechanisms of thrombotic APS that have thus far been explored in animal models will also be briefly addressed.

## 1. Introduction

Antiphospholipid syndrome (APS) is an autoimmune thromboinflammatory disease characterized by vascular thrombosis and obstetric complications in the setting of one or more antiphospholipid antibodies (aPL). With a prevalence of roughly 1 in 2000, a defining feature of APS is its ability to promote thrombosis in vascular beds of all sizes, including both arterial and venous circuits [1,2]. Thrombosis in APS remains insufficiently understood, and therapies targeting underlying pathophysiology are absent. About 70% of patients with APS experience peripheral thrombosis, with DVT being the most common thrombotic event [3]. In a smaller fraction of patients, thrombi form within microcirculatory vascular beds. In its severest form, microvascular APS can present as catastrophic APS (CAPS) characterized by multi-organ failure and high mortality.

Beyond thrombosis and pregnancy loss, APS regularly manifests with other morbid features including thrombocytopenia, cardiac valve destruction, accelerated atherosclerosis, nephropathy, movement disorders, and cognitive decline [4]. This heterogeneity of potential presentations highlights APS as a truly systemic autoimmune disease and underscores the need for a better understanding of disease mechanisms that will enable a personalized approach to management. Indeed, adjustment of antiplatelet, anticoagulant, and/or immunomodulatory medications is most often based on a reaction to another morbid event rather than a proactive attempt to prevent that event in the first place.

Despite the name of the syndrome (anti-phospholipid), the best-characterized autoantigen in APS is not a phospholipid, but rather a lipid-binding protein that circulates at high levels in the blood (100–200 µg/mL) known as apolipoprotein H (APOH) or beta-2 glycoprotein I (β_2_GPI). Autoantibodies to β_2_GPI activate various cell types in vitro [5,6,7,8] and promote both thrombosis and pregnancy loss when injected into mice [9,10]. Currently, three assays are used to classify a patient as having APS: *(i)* measurement of anticardiolipin antibodies, *(ii)* measurement of anti-β_2_GPI antibodies, and *(iii)* the lupus anticoagulant functional assay [11]. The latter detects various species of aPL based on their paradoxical prolongation of in vitro clotting times, including anti-phosphatidylserine/prothrombin (anti-PS/PT) antibodies [12].

While aPL circulate at relatively stable levels in the blood, thrombosis tends to manifest as discrete and acute events. It is assumed that the intravascular space is primed toward a prothrombotic state by aPL, but then a “second hit” is necessary to trigger the thrombotic event itself. While this two-hit model is generally accepted, much remains to be learned about how exactly aPL predispose to thrombosis in vivo, as well as how this predisposition interacts with the second hit.

In APS thrombosis models, injection of aPL (i.e., passive immunization) is typically used to induce an APS-like disease state. Antibodies (0.1–2 mg) have been administered via intraperitoneal (IP), tail vein (intravenous/IV), or retro-orbital (also IV) routes. The aPL may be administered before or after surgical intervention, and via a single injection or a series of injections. Both APS patient serum and serum from β_2_GPI-immunized rabbits have been used as sources from which to purify aPL; monoclonal antibodies have also occasionally been used. At this point, there are insufficient data to determine which antibody preparation or route of administration best replicates the APS disease state, and choices have been relatively siloed among different research groups.

Numerous approaches for modeling APS in animals have been described to date, each with potential advantages and disadvantages for characterizing aPL-mediated thrombosis. These animal models typically focus on a single type of vascular bed: namely venous, arterial, or microcirculatory. This review will attempt to describe the most common models employed to date within each category while discussing some advantages and disadvantages of each (Table 1). Mechanisms of thrombotic APS that have thus far been explored in animal models will also be briefly addressed.

## 2. Venous APS Models

### 2.1. Femoral Vein Pinch

**Figure 1:**
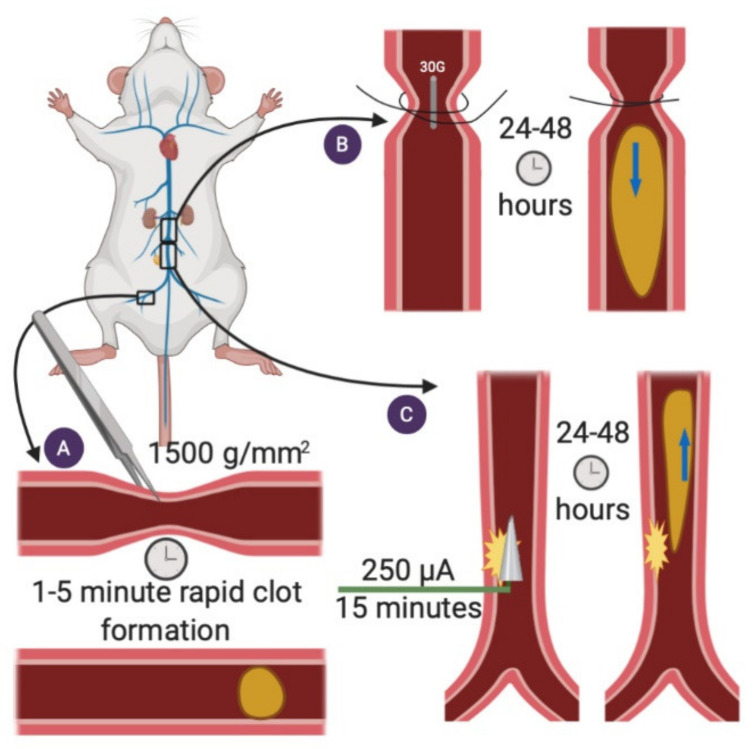
APS models of venous thrombosis. (**A**) Femoral vein pinch. A pinch injury is applied to the femoral vein, and rapid clot formation occurs 1–5 min following the injury [14]. (**B**), Stenosis. A ligature is placed around the IVC to achieve 90% occlusion, the spacer is removed and a thrombus forms in opposition to blood flow [15]. (**C**) Electrolytic IVC. Current is applied to a needle inserted into the IVC for 15 min. A thrombus grows in the direction of blood flow [20].

The femoral vein pinch model, first developed by Pierangeli and colleagues in 1994, was initially used to establish the relationship between high levels of aPL and clinical thrombosis [13]. This model applies a standardized pinch pressure to the femoral vein to generate multiple non-occlusive thrombi within the vein lumen (Figure 1A). Video taken during the experiment is used to measure the area of generated thrombi and time to thrombus formation and dissolution.

The surgery begins by excising the skin overlying the right femoral vein to expose a 0.5-cm segment of the vein. Next, a standardized “pinch” with a pressure of 1500 g/mm^2^ is applied to the vein to induce thrombosis. One minute after the pinch injury, a snapshot of the vein-thrombus interface is taken. Thrombus area is measured by tracing the outer margins of the clot in the digitized image. The time to thrombus formation and the time to thrombus dissolution may also be recorded [14].

The femoral vein pinch model is ideal for investigating thrombus propagation and dissolution because it enables real time visualization of thrombus progression. Given that the duration of the pinch injury may affect thrombus generation, laboratories must take care to have consistent pinch pressure application times to ensure standardized results.

### 2.2. Stenosis

The stenosis mouse model is well equipped to study the initiation of large-vein thromboses, such as deep vein thrombosis (DVT) in patients with APS (Figure 1B). In this model, a partial flow restriction (stenosis) is created in the inferior vena cava (IVC) to mimic blood flow stagnation in venous valves, a major cause of DVT in humans [15]. The duration of the stenosis model (typically 6, 24, or 48 h) can be adjusted to characterize different stages of DVT initiation [15]. Generated thrombi primarily form via laminar (non-turbulent) blood flow.

Under surgical anesthesia, a laparotomy is performed, the small bowel is exteriorized, and lateral branches of the IVC are ligated using 7-0 Prolene. Next, precise dissections are made caudal to the IVC and left renal vein junction to separate the IVC from the aorta. A 7-0 Prolene ligature is then fastened around the isolated IVC, using a blunted 30-gauge needle as a spacer to achieve roughly 90% vessel occlusion [15]. Following ligation, the spacer is removed, the abdomen is closed, and the mouse is allowed to recover. Mice are typically euthanized 6 to 48 h following surgery to assess thrombus formation. After excising the IVC, thrombi can be measured and then snap frozen or formalin-fixed for further analysis.

The stenosis model generates a thrombus that is structurally and histologically similar to human thrombi [15]. As the model generates thrombi in the absence of venous endothelial denudation [16], the stenosis model may be more relevant for studying DVT than mechanisms that induce thrombosis via endothelial damage, such as the ferric chloride model [16]. The stenosis model’s most significant limitation is variable thrombus size, which necessitates more mice per experimental group [17]. The cause of this variability is unknown, but it likely relates to anatomical differences among mice [18]. There is also debate regarding the extent to which patency of the infrarenal side branches is a key determinant of thrombus size [19].

### 2.3. Electrolytic IVC

The electrolytic IVC model is a venous thrombosis mouse model that produces a non-occlusive thrombus in the presence of continuous blood flow (Figure 1C). Applying constant direct current to a copper wire inserted into the IVC generates free radicals, which then activate endothelial cells and initiate thrombosis [20]. Similar to humans, thrombi produced in the electrolytic IVC model form in the direction of blood flow (unlike the stenosis model where the thrombus grows in opposition to flow). Thrombus weight is both current and time-dependent [21].

To perform the electrolytic IVC model, the IVC is exposed (similar to the stenosis model), and lateral branches are ligated using 7-0 Prolene suture, leaving any posterior branches patent. Next, a 25-gauge needle clamped to a 30-gauge silver-coated copper wire is inserted into the IVC and positioned against the anterior wall of the vessel (anode). Another needle (cathode) is implanted subcutaneously to complete the circuit. With both wires inserted, a constant current of 250 μA is applied for 15 min via a voltage-to-current converter. The needle is then carefully removed, pressure-induced hemostasis is achieved, and the abdomen is closed. Like the stenosis model, thrombi can be analyzed 6–48 h later. Other studies have investigated thrombus formation at longer time points, from 72 h to 2 weeks, albeit in a non-APS setting [22]. During thrombus isolation, the IVC is separated from the adjacent aorta, and the thrombus is trimmed of fat and vein wall. The isolated thrombus can be snap-frozen for Western blotting or formalin-fixed for immunohistochemistry.

The electrolytic IVC model produces thrombi of relatively consistent size across a variety of experimental time points [17]. This model may be ideal for investigating therapeutic agents, which due to the presence of continuous blood flow remain in contact with the thrombus. Challenges of this model include a longer operative time and damage to the IVC wall at the needle insertion site. Additionally, this model can lead to necrosis of the reproductive organs in female C57BL/6 mice [36]. For this reason, male mice are generally favored for the electrolytic IVC model, which results in sex bias [22].

### 2.4. Ferric Chloride Injury Model—Femoral Vein

The femoral vein ferric chloride (FeCl_3_) model is used to mimic venous thrombosis. Under anesthesia, the femoral vein is exposed and isolated from the femoral artery via a narrow incision in the upper inner leg of the mouse. Then, a filter paper soaked in FeCl_3_ is applied to the vein. After 1–5 min, the paper is removed, and the thrombus is allowed to develop within the vein lumen. Twenty minutes later, the thrombus is excised, measured, and processed based on laboratory preference [23].

Advantages of the femoral vein FeCl_3_ model include a straightforward surgical procedure and a brief time to thrombosis. A limitation of the model is the production of a transmural vein wall injury that may not replicate clinical DVT cases [17].

### 2.5. What Have We Learned about APS-Associated Venous Thrombosis from Animal Models?

Animal models have been central to understanding the role of various mechanistic factors in the pathogenesis of APS-mediated thrombosis (Table 2). Autoantibodies targeting domain I of β_2_GPI (anti-DI) are thought to be especially pathogenic, functioning as a better predictor of thrombotic risk in APS patients than either anti-β_2_GPI or anticardiolipin antibody levels [37,38]. The same seems to hold true in mice where injection with anti-DI-rich IgG generate larger venous thrombi as compared with mice injected with anti-DI-poor IgG [39]. Regarding mechanisms of thrombosis, these venous models have suggested that the exaggerated interplay between leukocytes and the endothelium is critical to APS pathophysiology. For example, disruption of endothelial adhesion molecules such as E-selectin and VCAM-1 (or their counterparts on leukocytes such as PSGL-1) reduces the size of aPL-mediated venous thrombi [40,41]. More recent work has suggested that beyond adhesion of neutrophils, the release of neutrophil extracellular traps (NETs) by activated neutrophils may be another therapeutic target [42]. NETs are prothrombotic webs of chromatin and microbicidal proteins released by dying neutrophils—a process that is over-exuberant in APS [43]. Indeed, depletion of neutrophils, dissolution of NETs by deoxyribonucleases, or prevention of NET release by adenosine receptor agonists and natural gingerols have all proven effective in mitigating APS-associated venous thrombosis in mice [44,45,46]. Other likely synergistic pathways implicated in these models include NADPH oxidase [47], TLR4-associated signaling [23,47], and the complement cascade [48].

## 3. Arterial APS Models

### 3.1. Ferric Chloride Injury Model—Carotid Artery

The FeCl_3_ injury model has been implemented in many different vasculature beds, including carotid arteries, femoral veins, and mesenteric vessels [24,25]. This section will discuss the arterial application of this model in mice.

After anesthetizing the mouse, a midline incision is created from the manubrium to the hyoid to expose the right jugular vein. Next, a platelet- or leukocyte-labeling agent may be administered into the jugular vein. The left sternocleidomastoid muscle is then retracted to visualize the carotid artery, and a 5-mm section is isolated from the nearby vagus nerve. A filter paper soaked in FeCl_3_ is then applied to the exposed artery for 1–5 min. Following removal of the FeCl_3_ paper, the artery is flushed with saline. Real time video, Doppler flow, and fluorescent intravital microscopy can be used to visualize blood flow, platelet aggregation, and clot formation from the onset of injury through euthanasia [24,25]. Thrombosis typically occurs within 30 min of the FeCl_3_ injury [25].

Strengths of the arterial FeCl_3_ injury model include the ability to visually track clot formation from onset of injury to full occlusion. As clots in this model are recovered just 30 min after initiation, this model eliminates the risk of death or complication during a lengthy recovery period, and it presents fewer logistical constraints. Disadvantages include a relatively difficult surgical procedure to isolate the carotid artery.

### 3.2. Photochemical Carotid

**Figure 2:**
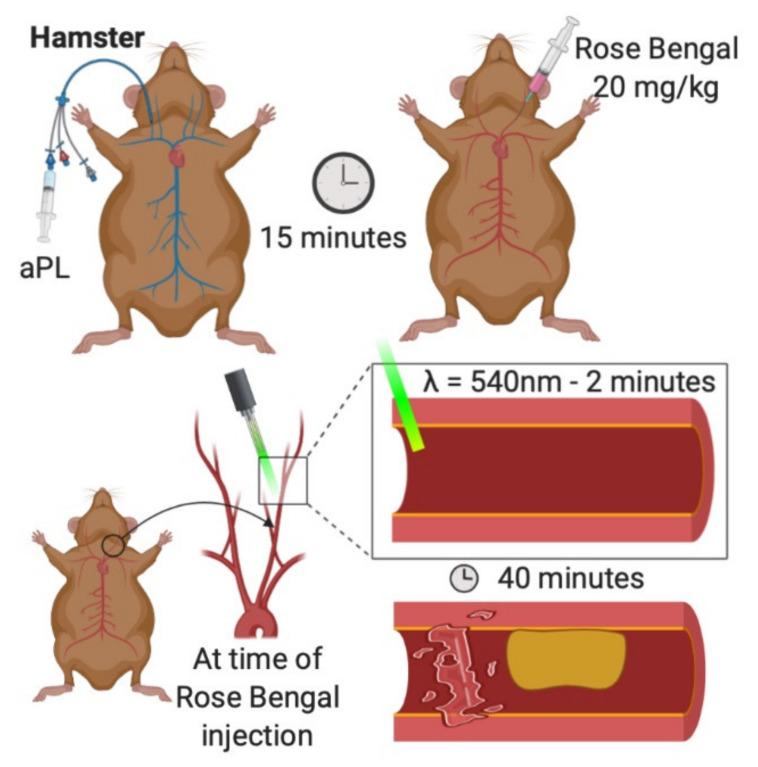
Photochemical carotid model of arterial thrombosis. aPL are injected into a jugular catheter. Rose Bengal is injected into the exposed carotid artery. The vessel is irradiated with green light for two minutes. Thrombosis is recorded over the next 40 min [26].

Photochemically induced carotid thrombosis has been used to simulate APS in hamsters [26] (Figure 2). Rose Bengal is a photosensitizing fluorescent dye that produces reactive oxygen species and focal vascular endothelial damage following exposure to green light. Beyond hamsters, the photochemical carotid model has been commonly used in vascular studies of thrombosis in other rodent species, including mice, although not to our knowledge in the context of APS [53,54,55].

Hamsters are first anesthetized with sodium pentobarbital [26]. Next, a 2.5 French venous catheter is inserted into the right jugular vein. The left carotid artery is then carefully exposed and mounted on a transilluminated stage. At this point, the carotid artery is injected with Rose-Bengal dye (20 mg/kg) and carefully irradiated for two minutes with green light (wavelength 540 nm) emitted from a xenon lamp. Thrombosis is visualized in the transilluminated carotid artery via intravital microscopy and is recorded (typically for 40 min). Snapshot images of the video may be digitized, and post-experiment analysis is typically performed by graphing transmitted light intensity versus time. Thrombus size and formation are assessed by calculating the area under the curve, expressed in arbitrary light units.

As described previously, reactive oxygen species contribute to platelet activation and thrombus formation [50]. Like the aforementioned FeCl_3_ and electrolytic models, the photochemical carotid model attempts to replicate endothelial dysfunction mediated by oxidative stress. A significant advantage to using a photochemical stimulus is the ability to standardize the degree of microvascular injury, producing highly reproducible results [56]. Catheterization of the animals in this model also allows for prompt intravenous delivery of experimental agents.

### 3.3. What Have We Learned about APS-Associated Arterial Thrombosis from Animal Models?

While the study of arterial vascular beds has not been as extensive as for venous, there are some notable similarities. TLR4 again appears to have an important role in APS-associated arterial thrombosis [23,49], as does the exaggerated formation of reactive oxygen species [57]. The pathogenicity of anti-β_2_GPI antibodies has been confirmed [26], with evidence that the antimalarial hydroxychloroquine may be an effective strategy for breaking the thromboinflammatory cycle [51].

## 4. Microvascular APS Models

### 4.1. Dorsal Skinfold Chamber

**Figure 3:**
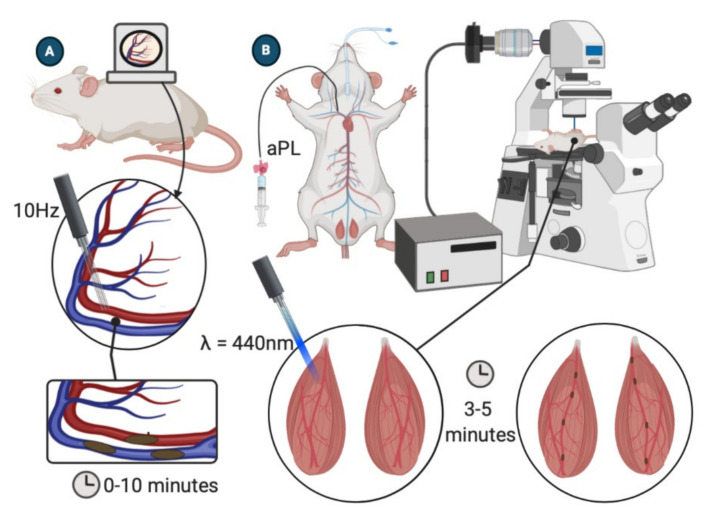
APS models of microcirculatory thrombosis. (**A**) Dorsal skinfold chamber model. Subcutaneous vasculature is visible through DCS window. A 10 Hz pulse laser s directed at the vessel wall for 30 s. Thrombosis is observed over the next 10 min [27,28]. (**B**) Laser-induced injury of the cremaster arterioles. Cremaster arterioles are exteriorized onto an intravital microscope tray. A laser is focused through the microscope objective and aimed at a selected vessel wall. Thrombosis is recorded via intravital fluorescent microscopy [29].

The Dorsal Skinfold Chamber (DSC) mouse model uses an implantable device to enable the long-term study of subcutaneous microcirculation (Figure 3A). This device provides visualization of the microcirculation during and after the prothrombotic induction of a laser injury [27]. The implantable nature of this device enables multi-day experiments.

The DSC model starts with shaving and depilating the back of an anesthetized mouse. The two sides of the chamber are implanted by sandwiching a double layer of cleared dorsal skin. Using a surgical microscope, a 15-mm diameter circle of dorsal skin is removed, and a coverslip is placed over the excised area and fixed to the chamber device. A 5–7-day recovery period after device implantation is required to reduce the influence of surgical trauma on the vasculature [27,28]. When ready for the experiment, mice are anesthetized, and intravital fluorescence microscopy is used to visualize blood flow in small cutaneous vessels through the dorsal skinfold chamber window. A 10 Hz pulse laser is then used to irradiate small vessels (50–80 μm in diameter) for 30 s, and the resulting thrombus formation is recorded for ten minutes. Multiple vessels may be irradiated in a single mouse [28].

The DSC model can provide data on thrombus size, as well as the time to thrombus formation and resolution. The chamber implanted in the mouse enables observation of the microcirculation for up to 3 weeks without adverse effects [27].

### 4.2. Laser-Induced Injury of Cremaster Arterioles

Thrombosis in the microcirculation is commonly investigated by laser-induced vessel wall injury of cremaster arterioles (Figure 3B). After anesthetizing the mouse, the scrotum is incised, and the testicle and surrounding cremaster muscle are exteriorized onto an intravital microscopy tray [29]. The cremaster is superfused throughout the experiment with a thermo-controlled and aerated (95% N_2_, 5% CO_2_) bicarbonate-buffered saline. Vessel wall injury is induced by a laser system (for example, Micropoint Laser System) focused through the microscope objective and aimed at the vessel wall. Intravital fluorescence data will typically be captured for 3 to 5 min following the injury [29,30,31]. The model can be repeated multiple times in a single mouse, inducing new thrombi upstream to avoid the influence of disrupted flow dynamics created by previously generated thrombi [32].

This model’s strengths include its ability to perform multiple thrombosis experiments in one mouse before and after treatment. Additionally, intravital fluorescence microscopy can provide valuable information on the kinetics of thrombus formation and the composition of resulting thrombi [29,30,32]. Other positive attributes of this model include a simple surgical protocol and a short procedure time. The model is limited by sex-bias, as female mice do not have cremaster muscles and cannot be used in these studies [29,30,31,32].

### 4.3. Ferric Chloride Injury Model—Mesenteric Microcirculation

The mesenteric FeCl_3_ model involves the application of FeCl_3_ over arterial and venous microvessels within the mesentery [25,51,52]. After anesthetizing the mouse, a laparotomy is performed, the intestines are exteriorized, and the mesentery is gently spread on a Petri dish to expose suitable vessels. Rhodamine 6G, a leukocyte and platelet labeling agent, is then injected intravenously (tail vein or jugular vein). The Petri dish is placed under an inverted microscope, and the chosen vessel is visualized. A FeCl_3_ soaked paper is then applied to the vessel, and thrombus formation is observed via fluorescence microscopy. After 1 min, the paper is removed, and the vessel is washed with saline. Dynamic thrombus formation continues to be monitored via the Rhodamine 6G-labeled platelets and leukocytes. Images are captured to measure the size of the resulting thrombi. Full occlusion of the vessel typically occurs within 30 min of removing the paper [24]. Several parameters, such as occlusion time, thrombus formation time, or thrombus size, can be investigated.

Thrombi formed by the FeCl_3_ model are highly sensitive to both anticoagulant and antiplatelet drugs, making the model well suited for the preclinical evaluation of new thrombolytic therapeutics [58,59]. Compared to the carotid artery FeCl_3_ model, the mesenteric model is less surgically intensive and easier to accomplish [24]. The mesenteric vessels are also easily accessible and are well suited for intravital microscopic observation of thrombosis. Regarding downsides, the reproducibility of the FeCl_3_ model can be limited by variable vessel size and the presence of visceral fat, which may shield the vessel from injury. Additionally, it is important to note that this model is not well suited for investigating endothelial inflammation-associated thrombosis, as it causes severe oxidative injury and endothelial denudation following FeCl_3_ application [24].

### 4.4. LPS-Priming

**Figure 4:**
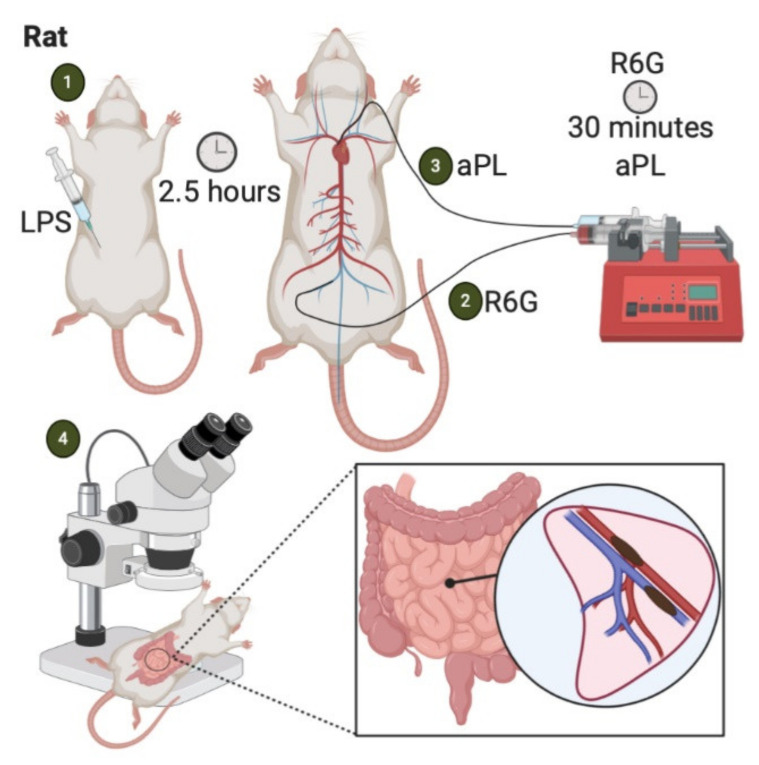
LPS-priming model of APS-associated thrombosis. (**1**) Intraperitoneal injection of LPS. (**2**) Two and a half hours later, Rhodamine 6G is infused into the femoral vein. (**3**) After 30 minutes, aPL are injected into arterial circulation. (**4**) Aggregates of fluorescently labeled platelets and leukocytes are observed in the mesenteric microcirculation via intravital microscopy [33].

The murine bacterial lipopolysaccharide (LPS) priming model is used to investigate APS-associated thrombosis within the mesenteric microcirculation [33] (Figure 4). In this model, a non-localized inflammatory stimulus, LPS, acts as the prothrombotic trigger rather than mechanical (pinch, stenosis)- or physicochemical (photochemical, FeCl_3_)-mediated endothelial injury.

Three hours before APS IgG infusion, Wistar rats are injected with an intraperitoneal dose of LPS or sterile saline as control. Two and a half hours later, the rat is anesthetized, and the left carotid artery and femoral vein are cannulated with polyethylene catheters connected to micro-infusion pumps. The carotid artery catheter tip extends to the aortic arch. A platelet/leukocyte-staining agent (Rhodamine 6G) is then gradually infused into the femoral vein. After 30 min, APS-IgG is promptly injected into the arterial circulation. At this point, intravital microscopy can be used to view fluorescent aggregates of leukocytes and platelets within mesenteric microvascular beds. Multiple microvascular areas that include arterioles, capillaries, and postcapillary venules may be examined to assess thrombus formation. An overall microvessel occlusion percentage is expressed by a ratio of total thrombi formed to total number of microvessels examined. Fibrin deposition in the endothelium can also be assessed with video-microscopy in this model by injecting an additional fluorescent labeling agent [33]. Finally, the mesenteric tissue can be harvested during euthanasia to enable immunofluorescent analysis of additional antibodies [33].

The LPS-priming model provides consistent thrombus generation and enables precise microvascular circulation analysis. Furthermore, because the LPS-priming model does not rely on a local vascular insult to induce thrombosis, this model might better mimic clinical thrombosis than models that employ chemical or physical damage [34]. Challenges of this model include a relatively complex surgical procedure and a prolonged operative time. Laboratories without the required surgical experience in intravital microscopy and catheterization may not find this a suitable model for investigating APS-associated thrombosis.

### 4.5. Histone Priming

The histone-priming model has been used to study the role of anti-PS/PT antibodies in APS [35]. Recent studies have shown that high titers of anti-PS/PT antibodies are a useful predictor of APS severity, while histones are likely important players in endothelial priming during inflammatory types of thrombosis [60,61]. Similar to the LPS-priming model, this model uses a non-localized inflammatory stimulus (i.e., cell-free histones) to induce thrombosis.

Wistar rats are administered an intravenous injection of calf thymus-derived histone (12.5 µg/g weight). Two hours later, they receive a second intravenous injection, this time with a rat anti-PS/PT monoclonal antibody (1.25 mg/g). After three days, rats are euthanized, and tissue sections of major organs are prepared for histopathology. One advantage of this model is its relative ease compared to its surgical counterparts; only two intravenous injections are required. Having said that, the non-surgical nature of the protocol prevents real time observation of thrombus formation. Additionally, this model can induce thrombosis in inconsistent locations, and therefore, may not be suited for studies of thrombosis in specific vascular beds. Furthermore, the relevance of the only monoclonal antibody studied to date to human APS remains unclear. Due to its relatively recent conception as an APS-thrombosis model, the capability of this model to explore other mechanistic features of APS pathogenesis is not yet known.

### 4.6. What Have We Learned about APS-Associated Microvascular Thrombosis from Animal Models?

A significant unmet need in clinical practice is the identification of effective approaches for the treatment of microvascular APS. From animal models, we have seen the potential for auto-amplifying crosstalk between platelets and endothelial cells when microvascular beds are bathed with aPL [30]. Dysregulation of the complement cascade [33] and nitric oxide synthase [52] also have the potential to be contributory in this context. Given that microvascular thrombosis in mice can be neutralized by therapeutics targeting the interaction between β_2_GPI and anti-β_2_GPI antibodies [31,33], it is perhaps not surprising that plasmapheresis (i.e., removal of anti-β_2_GPI antibodies) remains the most time-tested approach for the treatment of severe microvascular APS in humans [62].

## 5. Conclusions

In pursuit of targeted therapies for APS patients that may eventually minimize the need for life-long anticoagulation, animal models hold significant potential to unlock aspects of APS pathophysiology that could not otherwise be identified. Important opportunities for the future include more strategic attention to biological variables, including age and sex; confirmation of mechanistic discoveries in different models and across different vascular beds; and the establishment of synergistic partnerships between research groups that have complementary expertise. As the phenotyping of APS patients continues to deepen and new hypotheses are generated, we anticipate that animal models will remain an essential part of the preclinical exploration that will set the stage for a new era of APS clinical trials.

## Figures and Tables

**Table 1:** Strengths and limitations of various animal models of APS thrombosis.

Thrombosis Model	Strengths	Limitations
**Venous Models**		
Femoral Vein Pinch [13,14]	Well suited for the study of thrombus propagation and resolution; enables real-time visualization of thrombus formation.	Thrombus propagates against the direction of blood flow.
Stenosis IVC [15,16,17,18,19]	Variable thrombus generation enables study of thrombus initiation in prothrombotic conditions; thrombi produced are structurally similar to humans, thrombus experiences constant blood flow which supports the study of therapeutics; induces thrombosis without endothelial damage.	Variable thrombus size necessitates larger experimental group sizes; thrombus propagates against the direction of blood flow; challenging to observe thrombus formation in real-time.
Electrolytic IVC [20,21,22]	Thrombus experiences constant blood flow; suited for study of antithrombotic or thrombolytic agents; thrombi form in the direction of blood flow.	Longer operative time; physical damage to IVC vein lumen; can cause necrosis in the female reproductive organs of C57BL/6 mice; challenging to observe thrombus formation in real-time.
FeCl_3_ IVC [17,23]	Acute model suited to study early timepoints in thrombosis; thrombi form in direction of blood flow; intravital microscopy possible.	Smaller thrombus size can limit options for biochemical assays; transmural vein injury induced by FeCl_3_ may not mimic clinical thrombosis.
**Arterial Models**		
FeCl_3_ Carotid [24,25]	Acute model, suited to study early timepoints in thrombosis; thrombi form in direction of blood flow.	Requires a challenging surgical procedure to isolate the carotid artery; transmural vein injury may not mimic clinical thrombosis.
Photochemical Carotid [26]	Intravital microscopy enables real-time observation of thrombosis; catheterization allows easy administration of therapeutics; photochemical injury is highly standardizable.	Relies on local injury to produce thrombus; Acute model not suited for chronic studies.
**Microvascular Models**		
Dorsal Skinfold Chamber [27,28]	DSC device enables observation of the microcirculation for ≤3 weeks; real-time visualization of thrombosis initiation and resolution.	Requires surgery to implant DSC device and a recovery period before performing thrombosis experiments.
Laster-Induced Injury in the Cremaster Muscle [29,30,31,32]	Intraviral microscopy allows observation of thrombosis; easily accessible vascular bed; allows for the induction of multiple thrombi in the same mouse.	Can only be performed on male mice.
FeCl_3_ Mesenteric Microcirculation [24,25]	Easily accessible microvasculature; well suited for intravital microscopy; suited for acute study of thrombosis.	Variable vessel size and visceral fat can influence thrombus size; oxidative injury induced may limit applicability for studies of endothelial inflammation-associated thrombus.
LPS-Priming [33,34]	Consistent thrombus generation; real-time visualization of thrombosis with intravital microcopy; using a non-localized inflammatory stimulus, LPS, as a prothrombotic trigger might be more relevant to clinical thrombosis.	Complex surgical procedure; longer operative time.
Histone-Priming [35]	Has allowed study of anti-PS/PT antibodies in APS-mediated thrombosis; does not require surgical procedure.	Not suited for the study of thrombosis in specific regions; non-surgical procedure prevents real-time observation of thrombosis.

**Table 2:** Mechanisms of APS-associated thrombosis investigated using animal models.

Pathway/Factor	Role
**Venous**	
Anti-domain I	Pathogenic aPL bind the N-terminal domain of β_2_GPI (DI) [39].
Adhesion molecules	P-selectin, VCAM-1, PSGL-1, etc., facilitate interactions between leukocytes and the endothelium [40].
Deoxyribonucleases	Degradation of DNA (a key component of NETs) decreases thrombus size and incidence [44].
Adenosine receptor agonists	Adenosine receptor agonists (CGS21680, dipyridamole) suppress NETosis via stimulation of cAMP production [45].
Natural gingerols	Gingerols inhibit phosphodiesterase activity and suppress proinflammatory cytokine release [46].
NADPH Oxidase	NOX2-mediated tissue factor activation induces prothrombotic responses [47].
TLR4	The requirement for TLR4 may depend upon the type of aPL (anti-β_2_GPI versus cofactor-independent) [23,47].
Complement cascade	C3 in particular has been shown to be necessary for aPL-mediated thrombosis [48].
**Arterial**	
TLR4	TLR4 acts as a trigger of innate immune responses and has been shown to be necessary for aPL-potentiated thrombosis [49].
Reactive oxygen species (ROS)	ROS induce oxidative stress and exposure of subendothelial collagen, which promotes platelet adherence [50].
anti-β_2_GPI antibodies	Anti-β_2_GPI antibodies induce platelet activation [29].
Hydroxychloroquine	Hydroxychloroquine increases eNOS activity (other roles are also likely) [51].
**Microvascular**	
Platelet-endothelial cell interactions	Binding of anti-β_2_GP1/β_2_GP1 complexes to the developing thrombus activates platelets. Platelet-derived products then activate endothelial cells [30].
Complement cascade	Inhibition of membrane attack complex assembly protects against the prothrombotic effects of aPL [33].
Nitric oxide synthase	Increased levels of eNOS production potentiate NO production and thereby inhibit further platelet aggregation [52].
β_2_GPI/anti-β_2_GPI interaction	Disruption of the interaction between β_2_GPI and anti-β_2_GPI antibodies is protective [31,33].
